# Disparities in Quality of Park Play Spaces between Two Cities with Diverse Income and Race/Ethnicity Composition: A Pilot Study

**DOI:** 10.3390/ijerph120708009

**Published:** 2015-07-14

**Authors:** Gavin R. Jenkins, Hon K. Yuen, Emily J. Rose, Amy I. Maher, Kristina C. Gregory, Megan E. Cotton

**Affiliations:** Department of Occupational Therapy, School of Health Professions, University of Alabama at Birmingham, 1530 3rd Avenue South, Birmingham, AL 35294, USA; E-Mails: jenkinsg@uab.edu (G.R.J.); Emily80@uab.edu (E.J.R.); amymaher@uab.edu (A.I.M.); kkcrawfo@uab.edu (K.C.G.); cottonm@uab.edu (M.E.C.)

**Keywords:** built environment, environmental justice, parks, play value, play spaces

## Abstract

This study investigated the differences in the quality of park play spaces between an affluent and a non-affluent community in a large US Southeastern metropolitan area. Two cities were purposefully selected to reflect differences in household income and race/ethnicity characteristics. Using the Playable Space Quality Assessment Tool (PSQAT), all parks (n = 11, with six in the affluent city, and five in the non-affluent city) in these two cities were evaluated. The data were analyzed across three aspects of environmental features of the PSQAT: Location, Play Value and Care and Maintenance between parks in the two cities. The Mann-Whitney U test was used to test the study hypotheses. Results indicated significant differences between parks in the two cities in all three aspects of the PSQAT with *p*-values ≤ 0.03 and effect sizes of > 0.65, suggesting that the affluent city had parks of a higher quality than the non-affluent city. Significant disparity in Play Value (*p* = 0.009) in parks between these two communities suggests that children and young people are likely to have different experiences of the play spaces in their locality and therefore may experience different physical and psychological health benefits.

## 1. Introduction

Lack of access to safe and well-equipped city parks and public open spaces is more than an inconvenience. A longitudinal cohort study in Southern California demonstrated that limited access to parks and recreational resources was significantly associated with the development of childhood obesity [1]. Levels of physical activity in children and young people are influenced in part by access to safe parks and open spaces [2]. Communities that set aside greater proportions of space for park areas and ensure access to parks and recreational facilities have contributed to greater physical activity in young inhabitants [2,3,4]. In addition, specific park features such as amenities and playgrounds have also been shown to be associated with increased physical activity levels in children and young people [5,6,7,8].

More than 80% of Americans live in metropolitan areas [9], however, many residents of these cities lack adequate access to parks and open spaces near their homes [10]. Even in cities that have substantial park space, many neighbourhoods still have residents who lack access to near-by parks [10]. For example, in New York, nearly half of the city’s 59 community board districts have less than 1.5 acres (or 6070 square meters) of parkland per 1000 residents [11]. In addition, low income neighbourhoods, populated by minorities and immigrants, are especially short of city parks and public open spaces [12,13,14], and this inequitable distribution may be contributing to childhood and family obesity in these communities [4,15].

Although parks can provide an inviting setting to promote physical activity and active play, personal safety and quality of parks are major concerns for youths and parents [16,17,18,19]. Poor park maintenance, interracial conflict and discrimination, drug use, and gangs and crime rates in parks are some of the main reasons for low usage of parks in low socio-economic status (SES) communities [16,18,20].

Given the growing body of evidence as to the benefits of city parks and public open spaces to physical and mental well-being of residents in communities [21,22], the issue of environmental injustice in low-income and racial/ethnic minority communities becomes a focus of debate. Environmental injustice in the context of public open space and parks is defined as the disproportionate burden of accessing these local facilities, which enable people to lead healthy lives, experienced by economically and socially disadvantaged populations [23,24]. Researchers have studied how various income and racial/ethnic groups have different levels of access (availability and distribution) to parks [4,13,25,26]. Several studies have investigated the disparities in park quality between neighborhoods [27,28,29,30]. In general, they found the quality and condition of park features are unequal across areas comprised of varying income and race/ethnicity composition [27,28,29,30].

However, the audit assessment tools [31,32,33,34,35,36,37,38] that previous studies used to evaluate and quantify various environmental features and amenities including physical quality and condition (aesthetics and maintenance), proximity, incivilities, and safety of parks and open spaces do not consider social environment and play value for children and young people [39]. Research revealed that social environments, which provide opportunity for children and young people to share, negotiate, cooperate, resolve conflicts and learn self-advocacy skills, and play value of the environment are as important as the physical quality to encourage park use and park-based activity [39,40]. Play value in the context of parks and public open spaces refers to the usability of any environmental features or play areas as a setting for play, generating diverse (range and quality) play opportunities and experiences, which may include disability friendly and culturally relevant features, that suit various children's needs, motivations and abilities [41,42,43]. Such play opportunities and experiences support healthy growth and development in children and young people [44].

Play value itself is a rather abstract and subjective concept that is difficult to quantify [45]. In addition to having the play space that is accessible and appeals to physical activity, play value of parks depends on the quantity, quality, and diversity of park features (facilities, play equipment and materials), play space and landscaping [43,46].

For parks that have high play value they not only draw children and young people to visit, but also provide opportunities for a variety of play activities (social / physical / imaginative / cognitive / creative) and the possibility to allow children adapting park elements for their own play purposes, and supporting free exploration [44,45]. These characteristics of parks entice children and young people to return time and time again for repeat visits [42,47].

On the other hand, parks with low play value have physical and social barriers for play activities such as limited space of close mown grass fenced with chainlink and ponds that are not open to fishing or playing with remote control boats [48]. Such park environments serve only limited play purpose, with static rather than dynamic features of play equipment, and do not support children's daily requirements for physical activity [44]. Parks with low play value often lack environmental (bio)diversity features or loose play materials for manipulation [43].

To fill the research gap with regard to potential play value inequality in parks and public open spaces between communities of diverse SES and race/ethnicity, the purpose of this study was to quantify the difference in the quality of environmental features (*i.e*., Location, Play Value and Care and Maintenance) of park spaces offered between two different income level and racial/ethnic composition communities in a large metropolitan area in the Southeastern United States (US). It was hypothesized that there would be a significant difference in these environment features between the park spaces with respect to Location, Play Value and Care and Maintenance in affluent and non-affluent cities.

## 2. Methods

### 2.1. Study Sample

Two cities, Mountain Brook and Irondale, in the same county (Jefferson), Alabama, US were purposively selected to reflect differences in household income and race/ethnicity composition. They are both suburbs of Birmingham, Alabama. Information from the city managers who are responsible for parks and recreation areas of their respective cities, identified that there were six city-operated parks in Mountain Brook, and five in Irondale.

Based on demographic data available from the United States Census Bureau Survey [49], Mountain Brook was considered to be affluent as indicated by its well above median annual household income ($131,281) of the residents while Irondale was not affluent with the median annual household income ($50,157) of the residents below that of the general population households in the US ($53,046). According to the Birmingham News [50], Mountain Brook is one of the nation’s most prosperous communities and as such is the wealthiest city in the state of Alabama. Characteristics of the two cities, including race/ethnicity distribution, education level of the residents, median value of owner-occupied homes, median annual household income, percentage of person below poverty line, and number of persons per square mile are shown in Table 1 [49].

**Table 1:** Comparison of the characteristics of the two study cities, extracted from the United States Census Bureau data, 2013.

	Irondale	Mountain Brook
Population	12,349	20,413
Race/Ethnicity		
White alone, not Hispanic or Latino	54.0%	96.5%
Black alone	35.4%	1.0%
Hispanic or Latino	7.8%	1.0%
Asian alone	1.4%	0.9%
Native American alone	0.3%	0.1%
Education		
High school graduate or higher	87.4%	99.5%
Bachelor’s degree or higher	28.6%	85.4%
Median value of owner-occupied homes	$129,000	$542,800
Median annual household income	$50,157	$131,281
Persons below poverty line (percent)	15.7%	3.0%
Land area	17.3 sq mi	12.8 sq mi
Persons per square mile (sq mi)	713	1596
Number of city-operated parks	5	6
Persons per park	2470	3402
Proportion of land area to park	3.5 sq mi to 1	2.1 sq mi to 1

sq mi = square miles.

### 2.2. Procedures

From October 2014 to January 2015, four graduate students from the Master of Science Occupational Therapy program visited and evaluated all 11 parks in these two study cities. For practical and safety reasons the students divided into two teams to visit the parks in pairs on different time of the day and dates; and each student/assessor in the pairs carried out the evaluations independently using the Playable Space Quality Assessment Tool (PSQAT) for each park [39,51]. The assessors had completed a four-credit graduate course on Infants, Children & Adolescents in August, 2014. The objectives of this course are to provide students with an in-depth understanding and working knowledge on the following concepts which include the importance of play, stages of play development, types of play, function of play, identification of the values of various play experiences, play materials and play space, and administration of play assessments. In addition, the assessors received several hours of didactic training in the administration of the PSQAT prior to their visit to the parks. Two occupational therapy faculty supervised and undertook the analysis of the collected data and site reports.

### 2.3. Instrument

Incorporating the principle of successful play space [52], the INSPIRE Consultancy Ltd developed an audit assessment tool called “Playable Space Quality Assessment Tool” (PSQAT) which evaluates three aspects of environment features: Location, Play Value and Care and Maintenance of parks and public open spaces [39,51]. The ten principles for designing successful play spaces incorporated in the PSQAT to evaluate parks and playgrounds are: be ‘bespoke’ (custom-built play equipment designed especially for the site to meet visitors’ needs), be well located, make use of natural elements, provide a wide range of play experiences, be accessible to children with and without disabilities, meet community needs, allow children of different ages to play together, build in opportunities to experience risk and challenge, be sustainable and appropriately maintained, and allow for change and evolution [52]. Location includes items on how well children use play areas. Play Value considers the different, innovative and challenging ways that children have through a variety of play experiences. Finally, Care and Maintenance is related to items on safety quality as well as the degree that the play areas are free from unexpected hazards [51].

The PSQAT classifies play spaces and facilities into three different types: door-step (type A), local (type B), and neighbourhood (type C), according to the size, scale of the environmental features (facilities and amenities), and distance from neighborhoods. Type A facility is a small space, within sight of home, where children can play within view of their caregivers, and type B or C facility is a larger space that children can get to independently and safely with their peers [51].

Of the three sections in the PSQAT, there are seven items in the section Location (whether the site has informal oversight, is easy to get to, has been well used, provides opportunities to meet other children, and has attractive play space, and visitors feel safe), 10 items in Play Value (whether the site entices children to play, includes children with disabilities, meets the needs of different age groups, allows for movement, imaginative play, ball games and natural play with loose parts, and has places to sit), with two items not applicable to type A play space and facility, and seven items in Care and Maintenance (whether the site looks well maintained, has health and safety checks conducted regularly, seating for adults, litter bins, dog free zones, has supervisory adults, toilets and changing facilities), with two items not applicable to type A play spaces and facilities (see Table 2). Each item was scored in a six-point scale, ranging from 0 = absent, 1 = serious weaknesses, improvement needed, 2 = weaknesses, improvement needed, 3 = average, needs some improvement, 4 = good, to 5 = excellent. A missing score was assigned to items that were not applicable to the type of play space and facility of a particular park. A summation score of each section was formed by adding all items together. The maximum score of each section for type A play space and facility is: 35 for Location, 40 for Play Value, and 25 for Care and Maintenance. The maximum score for type B or C play space and facility is: 35 for Location, 50 for Play Value, and 35 for Care and Maintenance. The PSQAT also takes into account weather and the time the assessment took place that may inadvertently affect the scoring.

The PSQAT was used to assess the quality of all the children’s play areas of the 11 parks in Mountain Brook and Irondale. All park assessments were conducted during weekdays between 10:15 am and 4:30 pm with the weather considered to be either mostly sunny or sunny.

**Table 2:** Playable Space Quality Assessment Tool scores on park quality of the two study cities.

Section	Irondale (n = 5)			Mountain Brook (n = 6)		
	Median	Mean	SD	Median	Mean	SD
**Location**						
Informal oversight	3.00	2.55	0.97	4.50	4.33	0.75
Well used by children	2.50	2.10	1.08	4.88	4.38	0.93
Getting there	3.00	2.70	1.19	3.50	3.75	0.50
Personal safety, lighting and security	3.00	3.00	0.47	3.63	3.92	0.68
Accessibility in getting there	2.75	2.15	1.05	3.00	3.00	0.79
Meeting other children	2.50	2.45	1.10	4.13	4.13	0.79
Designed for the site	1.50	1.85	0.78	4.00	3.21	2.11
**Play Value**						
Enticing to children to play	2.50	2.30	1.04	4.00	4.13	0.31
Play needs of different ages (B & C only)	3.38	3.19	0.47	5.00	3.60	2.19
Movement	2.00	2.30	0.78	3.50	3.58	0.86
Imaginative play ages (B & C only)	2.00	2.00	0.46	2.75	3.15	1.27
Ball games	2.75	2.60	1.61	1.63	2.54	1.64
Loose parts	1.00	1.05	0.11	1.88	2.13	0.70
Access to the natural environment	2.50	2.55	0.45	3.50	3.88	0.70
Places for children to sit	3.50	3.50	0.77	4.13	4.00	0.65
Play opportunities for disabled children	1.00	1.20	0.27	2.25	2.21	0.73
Added play value	1.25	1.25	0.25	3.00	2.83	1.00
**Care and Maintenance**						
Well maintained	3.00	2.95	0.84	5.00	5.00	0.00
Health and safety	2.50	2.60	0.42	4.63	4.54	0.46
Seating for adults	4.00	4.10	0.74	5.00	4.67	0.82
Litter bins	4.25	4.20	0.78	5.00	4.96	0.10
Dog free zones	2.00	2.30	1.05	2.00	2.88	1.66
Presence of supervisory adults (B & C only)	1.50	2.25	1.69	3.00	2.75	1.39
Toilets / changing facilities (B & C only)	1.38	1.69	1.70	1.00	1.95	1.37

### 2.4. Data Analysis

The intra-class correlation coefficient was used to estimate the inter-rater reliability among the four assessors evaluating each of the 11 parks. Cronbach’s alpha was used to estimate the internal consistency reliability of items within each of the three sections of the PSQAT. Association between the summed scores of the three sections of the PSQAT was determined by the Spearman's rank-order correlation.

The summed scores of each section of the PSQAT were converted into percentage scores to allow for weighting and comparisons between the Location, Play Value and Care and Maintenance sections within the 11 parks and between the parks of the two cities. Since the percentage scores of the three sections did not meet the assumptions of normality, non-parametric statistical methods were used to analyse the data. Specifically, the Wilcoxon signed ranks test was used to evaluate significant difference in the percentage scores between the three sections of the PSQAT within the 11 parks. The Mann-Whitney U-test with exact p-values was used to evaluate significant difference of the percentage scores of the three sections (Location, Play Value and Care and Maintenance) of the PSQAT between the parks of the two cities. All hypothesis tests were based on a two-sided alpha of 0.05. All data analyses were conducted using the IBM Statistics Package for Social Sciences (SPSS) for Windows, version 22 (www.spss.com).

## 3. Results

The median, mean, and standard deviation scores of individual items within each section of the PSQAT for the parks in the two study cites are shown in Table 2.

### 3.1. PSQAT Reliability Assessment

For inter-rater reliability, the intra-class correlation (ICC 2,1) coefficient among the four assessors for Location was 0.95 (95% confidence interval [CI] = 0.87 to 0.99), Play Value was 0.86 (95% CI = 0.68 to 0.96), and Care and Maintenance was 0.88 (95% CI = 0.75 to 0.96). All ICC coefficients were above 0.85 which indicated excellent inter-rater reliability among the assessors [53,54].

The internal consistency reliability of the three sections (Location, Play Value and Care and Maintenance) of the PSQAT as estimated by Cronbach’s alpha was 0.9 (95% CI = 0.76 to 0.97), 0.80 (95% CI = 0.53 to 0.95), and 0.81 (95% CI = 0.53 to 0.95), respectively which was considered to be good to excellent [55]. Significant correlations were observed among the percentage scores of the three sections of the PSQAT (ps ≤ 0.001), with all three correlation coefficients (Spearman’s rho) ≥ 0.85.

### 3.2. Comparisons of Scores between the Three Sections of the PSQAT within 11 City Parks

Percentage scores of Play Value were observed to be systematically lower than those of the other two PSQAT sections (Location and Care and Maintenance) across all 11 parks (see Table 3). Based on the analysis from the Wilcoxon signed ranks test, significant differences were observed between the median ranks in percentage scores of Play Value and Location (Z = −2.67, *p* = 0.008) and Play Value and Care and Maintenance (Z = −2.67, *p* = 0.008) in the two cities’ parks.

### 3.3. Comparisons of the PSQAT Scores between Parks of the Two Study Cites

Significant differences in the median percentage scores of the three sections of the PSQAT between parks of the two cities were observed at the 5% level of significance (see Table 3). The *p*-value for Location, Play Value and Care and Maintenance was 0.03, 0.009, and 0.03, respectively. The effect size for Location or Care and Maintenance between parks of the two cities was 0.66, and the effect size for Play Value was 0.77, both values are considered medium effect [56,57].

**Table 3:** Comparison of the summed scores in percentage of the three sections of the Playable Space Quality Assessment Tool on park quality of the two study cities.

Section	All (n = 11)			Irondale (n = 5)			Mountain Brook (n = 6)					
	Median	Mean	SD	Median	Mean	SD	Median	Mean	SD	U		*p*-value
**Location**	62.86	63.44	19.53	47.14	48.00	14.06	82.14	76.31	12.94	3.0		.03
**Play Value**	55.00	54.18	13.73	43.50	43.10	8.97	61.00	63.42	9.35	1.0		.009
**Care and Maintenance**	68.57	68.77	15.99	53.57	58.11	14.21	76.43	77.64	11.91	3.0		.03

SD = standard deviation, U = Mann-Whitney U statistic.

## 4. Discussion

Even though the ratio of persons to park in Irondale is lower than in Mountain Brook as shown in Table 1, the overall park quality of Irondale is inferior to that of Mountain Brook. Results from the present pilot study indicated there were disparities in park access and quality between these two communities of diverse income levels and race/ethnicity composition, which are consistent with findings reported in the literature [27,28,29,30]. Significantly lower scores across the three aspects of environment features (Location, Play Value and Care and Maintenance) of park spaces were found in the non-affluent community (*i.e*., Irondale). In other words, children in the affluent community (*i.e*., Mountain Brook) potentially have access to parks and recreation areas that are of a higher quality than in the community (*i.e*., Irondale) with lower SES.

The location of areas for play and recreation is perhaps the single most important factor in determining how well children and young people use these parks [51]. As indicated by persons per park, land area, and proportion of land area to park between the two study cities in Table 1, the issue of disparities in Location does not seem to be attributed to the actual physical location accessibility, rather the concerns are on the inferiority in informal oversight, and personal safety, lighting and security of parks in Irondale. With the increasing tendency of parents to limit the physical play space range of children and young people, having safe local play places is more likely to ensure use and participation [47,58].

The second aspect of environmental features evaluated through the PSQAT was Play Value. Play Value focuses on the different, innovative and challenging ways in which play areas can offer a variety of opportunities and experiences to suit various children’s needs, motivation and abilities [39,41,42,51]. Play spaces offer many varied and interesting opportunities and the results of this study indicated that appropriate and stimulating play environments are more likely to exist in a community of higher SES than a community of lower SES. For children in the affluent community, the variety of play experiences are wider than those found in play spaces located in the non-affluent community. Future studies may seek to quantify the nature and types of play and recreation that children and young people engage in across diverse play spaces, as well as to explore the physical and psychological health benefits resulting from the more enriched play experience offered in parks with higher play value.

The final aspect of environmental features that the PSQAT focuses on is Care and Maintenance. All children and young people need spaces that are free from unexpected hazards. Assessing the quality of care and maintenance of the play spaces in the two study cites showed that the affluent community was more likely to be able to allocate resources over time to maintain the area free of unexpected hazards. This appears understandable as care and maintenance requires on-going budgetary commitment, which may be more difficult to sustain in a non-affluent community.

The results showed an overall difference in quality of the three aspects of environment features assessed by the PSQAT, indicating that the investment strategies adopted by the City of Mountain Brook supports the quality of play spaces more than Irondale. It seems the difference in the park quality of these two cities could be due to the fact that Mountain Brook (the more affluent city) collects more tax money, and thus can spend more on parks, including staffing and resources. According to the statistics from City-Data (www.city-data.com/.com), in 2012–2013, the median real estate property taxes paid for housing units was about $5,000 in Mountain Brook *vs.* $800 in Irondale, with six times more in Mountain Brook. The operation budget assigned to parks and recreation was about 2.6 million dollars in Mountain Brook *versus* eight hundred thousand dollars in Irondale. In addition, the City of Mountain Brook employed 14 full-time and one part-time staff persons in the Department of Parks and Recreation, whereas the City of Irondale employed only four full-time staff persons.

Knowing the differences in the quality of parks offered by these two cities, planners and communities could leverage them by better allocating resources and environments to enhance the quality and use of these vital community spaces [59], thus supporting the physical and psychological well-being of the young residents. Policy makers and legislators need to understand the nature of disparities related to various park characteristics and features in these two cities. Findings from this study may assist the development of policy and standards regarding appropriate modifications within city parks and public open spaces leading to the promotion of park use, and park-based activity for children and young people at the community level. However, unless there is a metropolitan or state authority that can allocate resources for parks across these two cities, or non-profit organizations, like the Trust for Public Land, that can help the community raise funds, and renovate the parks and playgrounds in Irondale, the park inequality between these two cities will remain. Finally, given that the Play Value of parks in both communities was inferior in two other aspects of environmental features (*i.e.*, Location and Care and Maintenance) of park quality, investment of more resources is recommended in environment features related to play value when it comes to budgetary allocation on improving park quality.

### Limitations

Given the concerns about the small number of parks and purposive sampling of the two cities, we acknowledge that parks audited in this study may or may not represent parks in affluent and non-affluent cities of Alabama; therefore, caution should be exercised regarding generalization of study results. Confirmation of results reported in the present study should come with a population study in which a stratified random sampling of cities in Alabama with SES levels as the stratum. The PSQAT was developed based on a growing body of knowledge about good design principles in outdoor play and about where and how children like to play (*i.e*., strong content validity). Results from the inter-rater reliability, internal consistency, and correlations between sections of the PSQAT also indicated that PSQAT demonstrated strong psychometric properties of reliability. However, structural validity, criterion validity, cross-cultural validity and responsiveness of the PSQAT need to be established. For example, longitudinal studies with larger samples are needed to establish the validity of PSQAT for predicting physical and psychological well-being over time.

## 5. Conclusions

Equitable access to quality play spaces that allows children and young people to take risks, to explore and to be challenged, is an important part of childhood development. This study quantified the difference in the quality of environmental features (*i.e*., Location, Play Value and Care and Maintenance) of park spaces offered between an affluent and a non-affluent community in the Birmingham metro area, Alabama. Results showed an overall difference in quality of the three aspects of environment features as assessed by the PSQAT. Significant disparity in Play Value in parks between these two communities suggested that children and young people are likely to have different experiences of the play spaces in their locality and therefore may experience different physical and psychological health benefits. This study provides a further window into the challenges of providing suitable play environments and opportunities for children and young people who live in low income neighborhoods, populated by minorities and immigrants.
